# Linking clinical and imaging diagnostic assessments of the feline hypertrophic cardiomyopathy phenotype

**DOI:** 10.3389/fvets.2025.1720886

**Published:** 2025-12-18

**Authors:** Felipe Gaia de Sousa, Ruthnea Aparecida Lazaro Muzzi, Roberto Baracat de Araújo, Rafael Resende Faleiros, Fabiana Silva Fádel Queiroz, Suzane Lilian Beier

**Affiliations:** 1 Department of Veterinary Clinic and Surgery, Veterinary School, Federal University of Minas Gerais - UFMG, Belo Horizonte, Minas Gerais, Brazil; 2 VETHEART – Cardiovascular Physiology and Veterinary Cardiology Research Group, Belo Horizonte, Minas Gerais, Brazil; 3 INCT Nanobiofar – National Institute of Science and Technology in Nano-Biopharmaceuticals, Belo Horizonte, Minas Gerais, Brazil; 4 Department of Veterinary Medicine, Faculty of Animal Science and Veterinary Medicine, Federal University of Lavras - FZMV/UFLA, Lavras, Minas Gerais, Brazil

**Keywords:** concentric ventricular hypertrophy, congestive heart failure, cardiovascular repercussions, feline cardiomyopathies, prognostic parameters

## Abstract

Hypertrophic cardiomyopathy (HCM) phenotype represents the most commonly diagnosed cardiac disorder in felines, characterized by heterogeneous clinical presentations and a well-established genetic basis. This study aims to integrate clinical, laboratory, and imaging diagnostic assessments of the feline HCM phenotype, providing a comprehensive perspective on how complementary diagnostic approaches enhance disease understanding and precision. The HCM phenotype is defined by concentric hypertrophy of the left ventricular free wall and/or interventricular septum, often accompanied by secondary left atrial remodeling due to chronic pressure and volume overload. Clinical signs typically emerge with disease progression, frequently culminating in congestive heart failure (CHF) and respiratory signs; however, some cats may remain asymptomatic. Accurate diagnosis of the HCM phenotype requires an integrative approach combining thorough clinical evaluation and advanced imaging modalities to avoid misdiagnosis, which may negatively impact prognosis and quality of life. Detailed clinical history and physical examination are essential for diagnostic orientation, particularly in symptomatic patients. Routine laboratory tests support systemic assessment, although no pathognomonic biomarker has been identified to date. Cardiac biomarkers such as atrial natriuretic peptide (ANP), N-terminal pro–B-type natriuretic peptide (NT-proBNP), and cardiac troponin I (cTnI) provide complementary diagnostic information, albeit with lower sensitivity than imaging techniques. While electrocardiography may reveal conduction disturbances suggestive of HCM, transthoracic echocardiography remains the diagnostic gold standard. In addition to confirming the diagnosis, echocardiographic evaluation allows for disease staging, longitudinal monitoring, and evidence-based therapeutic decision-making. Our study reinforces the need for an integrated diagnostic framework that combines clinical examination, laboratory testing, and imaging evaluation. By promoting a multidimensional diagnostic perspective, this study contributes to refining the understanding of the feline HCM phenotype and supports the development of more precise diagnostic and therapeutic strategies, ultimately improving clinical outcomes in affected cats.

## 1 Introduction

Cardiovascular diseases encompass a spectrum of clinical conditions commonly associated with deleterious pathophysiological processes that negatively affect both quality of life and overall survival. These disorders may be congenital, acquired, or degenerative in nature. In felines, acquired cardiac conditions are diagnosed more frequently than degenerative ones. Feline cardiomyopathies comprise a group of myocardial diseases occurring in the absence of identifiable systemic causes (1). Classification is based on phenotypic expression, emphasizing structural and functional characteristics (2). Beyond the hypertrophic phenotype, cats may present with dilated (DCM), restrictive (RCM), arrhythmogenic/arrhythmogenic right ventricular cardiomyopathy (AC/ARVC), or an unclassified phenotype (2). The DCM phenotype is marked by systolic dysfunction and global atrioventricular dilation (2). The RCM phenotype includes an endomyocardial form with apical obstruction and a myocardial form with normal ventricular size but atrial enlargement (2). AC/ARVC is characterized by right-sided dilation, reduced wall thickness, and systolic dysfunction (2). The unclassified phenotype encompasses cats whose features do not fit into a specific category (2).

Hypertrophic cardiomyopathy (HCM) is the most commonly identified phenotype in felines, accounting for 15%−78% of cases. It is characterized by concentric hypertrophy, primarily affecting the left ventricle (LV), usually resulting from alterations in sarcomeric proteins and thickening in the absence of increased afterload (2–9). In certain cases, cardiomyocyte injury accompanied by collagen fiber deposition occurs, leading to pressure overload (2–9). Although the LV is primarily affected, the right heart chambers can also be involved, with changes such as ventricular remodeling and dysfunction often observed and associated with increased disease severity (10–12). A well-documented breed and age predisposition exists, with higher prevalence in Maine Coon and Ragdoll cats, as well as in animals older than 6 years (3, 7, 8, 13–19).

The HCM phenotype can arise from primary or secondary causes. The primary HCM phenotype occurs when injury affects cardiomyocytes due to genetic alterations, leading to amino acid substitutions that impair sarcomeric function and result in structural and/or functional changes (20, 21). The genetic involvement is underscored by the identification of sarcomeric mutations that alter protein conformation (20, 21). Grzeczka et al. (22) suggest that high disease penetrance in homozygous individuals may indicate a toxic proteome as a pathogenic mechanism. Recent findings by Raffle et al. (23) identified sarcomeric variants, including cardiac α-actin (ACTC1), α-actinin 2 (ACTN2), MYH7, and TNNT2. Furthermore, another variant was identified, namely cysteine and glycine-rich protein 3 (CSRP3), a non-sarcomeric gene that may represent a potential biomarker in cats affected by HCM and restrictive cardiomyopathy (23). These variants support the hypothesis of a genetic overlap between these phenotypes (23). The genetic mutations currently identified lead to structural and functional sarcomeric alterations, thereby compromising myocardial function. Alternatively, it is important to note that the HCM phenotype can also be secondary to certain diseases or clinical conditions, such as hyperthyroidism, valvular stenosis, or systemic hypertension, for example (2). Therefore, it is essential to investigate the underlying cause of the HCM phenotype. Another possibility that may be observed and should be considered as a differential diagnosis is transient myocardial thickening, which is characterized by a temporary hypertrophic presentation that mimics the HCM phenotype (24, 25). This condition may be associated with a prior event and usually resolves spontaneously (24, 25).

Clinical manifestations vary considerably, although congestive heart failure (CHF) and its sequelae are common endpoints. Arterial thromboembolism (ATE) is also an occasionally reported complication, attributed to Virchow's triad and/or atrial blood stasis (2, 3, 6, 9, 17, 26). Diagnostic options include laboratory testing, particularly of cardiac biomarkers of myocardial injury and function, as well as other methods such as electrocardiography and transthoracic echocardiography (2, 5, 6). According to Sousa et al. (27), “various factors contribute to diagnostic challenges, including phenotypic overlap, difficulties in classification, and a high proportion of asymptomatic cases.” This study aims to establish the relationship between clinical findings and imaging-based diagnostic parameters in cats with the HCM phenotype. Furthermore, it aims to provide an integrated perspective on how these complementary assessments contribute to a more accurate characterization and understanding of the disease presentation.

## 2 Feline hypertrophic cardiomyopathy phenotype

**Figure 1:**
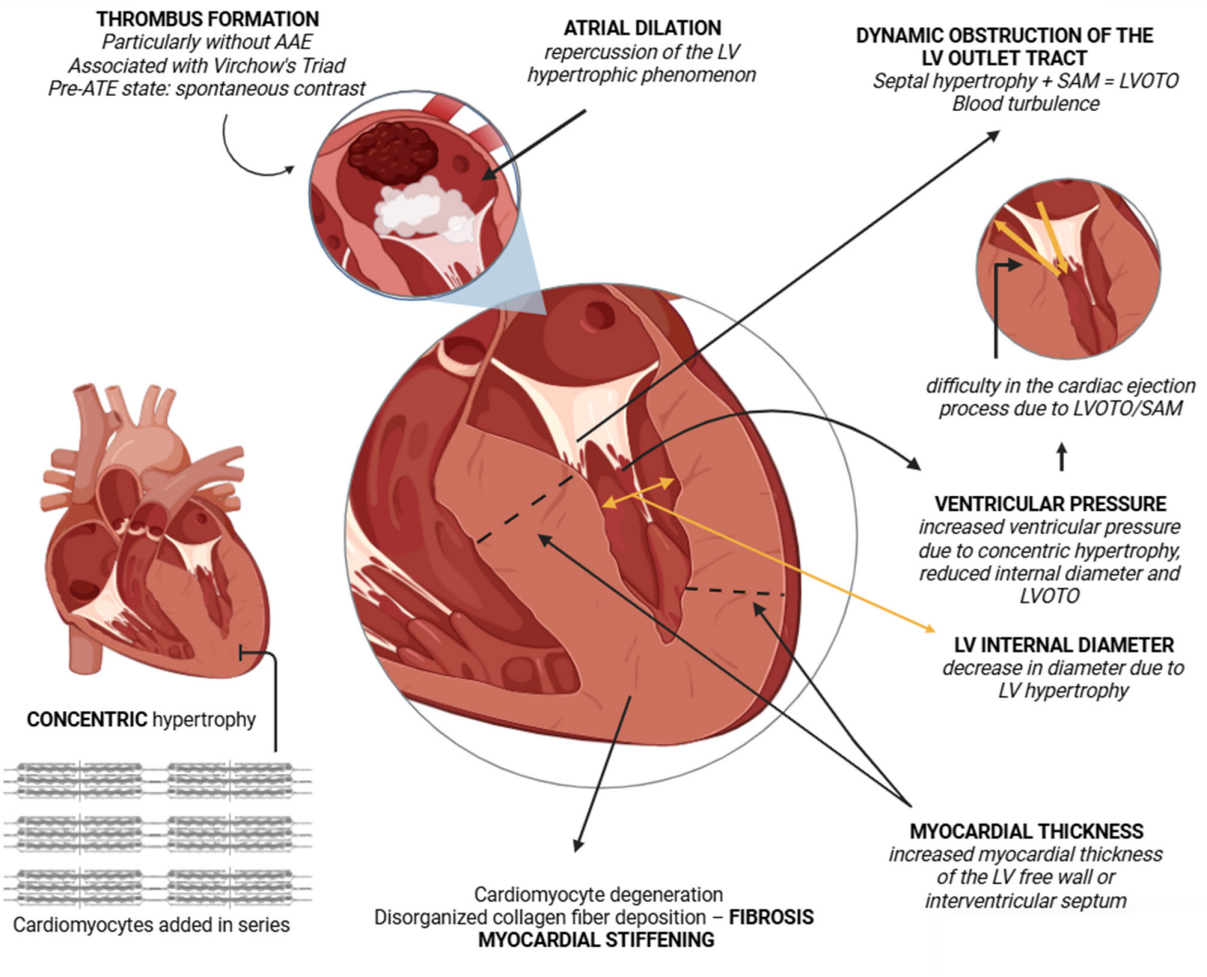
Schematic representation of the pathophysiology of the feline hypertrophic cardiomyopathy (HCM) phenotype. Note the presence of asymmetric hypertrophy, particularly in the septal region adjacent to the left ventricular outflow tract. Associated with elevated ventricular pressure, systolic anterior motion (SAM) of the septal mitral valve leaflet may occur, leading to increased pressure gradients, clinical worsening, and disease progression. Created by the authors with BioRender.com.

The HCM phenotype is defined by concentric hypertrophy involving the free wall or septal region, predominantly affecting the left ventricle (LV; Figure 1) (2, 3, 5, 6, 15, 17, 26, 27). This hypertrophy may present as symmetric or asymmetric, particularly when involving the LV outflow tract region (6, 28, 29). Although asymmetric involvement of the LV outflow tract region is common, the obstructive form of the disease is defined by dynamic functional factors, such as systolic anterior motion (SAM) and the resulting pressure gradient (6, 28, 29). Disease severity often correlates with pathological findings and clinical consequences, varying from mild to severe and directly proportional to its impact on hemodynamic function (2, 3, 6, 12, 14, 15, 26, 30).

The presence of diastolic dysfunction impairs ventricular relaxation; thus, in addition to structural alterations, there is a significant functional impact (Figure 1). Affected cats frequently exhibit reduced LV internal diameter, atrial dilation, and chronic activation of compensatory systems such as the sympathetic nervous system and the renin-angiotensin system (RAS) (5–7, 12, 31). The role of RAS in HCM pathophysiology was demonstrated by Lean et al. (32), who identified overexpression of angiotensin-converting enzyme 2 (ACE2) in affected felines, suggesting that increased ACE2 expression may potentiate disease progression, but the underlying mechanisms remain unclear. One of the consequences of HCM is ATE, correlated with Virchow's triad: blood stasis (e.g., LA stasis), endothelial injury, and hypercoagulability (Figure 1) (2, 33–37). In some cases, the ATE condition may represent the first manifestation of the HCM phenotype.

Clinical signs are heterogeneous and often linked to disease progression, with most affected animals exhibiting cardiovascular manifestations alongside respiratory symptoms (2, 6, 8, 14, 17, 30). It is well-established that clinical signs correlate with factors such as disease progression, breed, and age, with severity being the primary determinant of symptom onset. Common clinical findings in cats with HCM include tachycardia, tachypnea, cyanosis, exercise intolerance, dyspnea, syncope, gallop sound and/or murmurs (7, 12, 15, 16, 29, 39–47). In some cases muffled cardiopulmonary auscultation with crackles, pleural effusion, pulmonary edema, hypothermia, and signs secondary to ATE such as hind limb paresis and pain (7, 12, 15, 16, 29, 38–47).

## 3 Diagnostic evidence

### 3.1 Clinical evaluation and differential diagnoses

Initial feline assessment must include a thorough physical examination and detailed anamnesis focusing on signs suggestive of cardiac disease. Veterinary evaluations in cats should adhere to Cat Friendly Practice guidelines (48). Clinical examination enables systemic health assessment, with particular emphasis on cardiopulmonary evaluation and accurate, protocol-driven blood pressure measurement, often overlooked in practice (49–51). Situational hypertension mediated by stress or secondary causes is common in felines, necessitating identification and control of associated factors before diagnosis of arterial hypertension (2, 51). Given the risk of ATE, limbs and paw pads should be carefully inspected for pulse quality, coloration, and temperature (44) and other manifestations with pain and vocalization. Cardiopulmonary auscultation may be complicated by purring; placing a hand on the ventral larynx can facilitate the auscultation process (47).

Potential differential diagnoses must be excluded through clinical and laboratory evaluations. Important differentials include some diagnoses such as feline systemic hypertension, hyperthyroidism, valvular stenosis, acromegaly, pseudo-hypertrophy due to hypovolemia/reduced circulating volume, and, less commonly, angioendotheliomatosis. Another important differential diagnosis for feline myocardial thickening is cardiac infiltrations such as neoplasia/lymphoma, as reported in a case where the neoplastic infiltration mimicked decompensated HCM (52). The coexistence of chronic kidney disease (CKD) and systemic arterial hypertension can also contribute cardiac hypertrophy, but less frequently (6, 49, 53–56). Angioendotheliomatosis, characterized by intravascular proliferation of endothelial cells, has been described as a differential diagnosis due to myocardial involvement and clinical presentation mimicking stage B1 HCM phenotype, but is a unusual condition (55). However, hyperthyroidism is an important differential diagnosis because it can induce hypertrophic changes (57). In this case, measurement of thyroxine (T4) is recommended in cats diagnosed with HCM to rule out or confirm hyperthyroidism (5, 8, 58). Lee et al. (59) reported a case of a Persian cat presenting with lethargy, apathy, tachypnea and tachycardia, diagnosed with hyperthyroidism. Cardiac evaluation revealed cardiac hypertrophy, which regressed following pharmacological thyroid management. However, the association between hyperthyroidism and the HCM phenotype remains incompletely elucidated.

Owing to the unclear relationship between hyperthyroidism and the HCM phenotype, Janus et al. (54) conducted a necropsy-based comparative study between cats with hyperthyroidism and those with the HCM phenotype, with objective to elucidate the possible relation between them. Cats with HCM have increased transverse and longitudinal LV diameters, larger ventricular surface area, atrial appendage enlargement, symmetric and asymmetric hypertrophy, cardiomyocyte degeneration, and significant myocardial fiber disorganization. Conversely, hyperthyroid cats showed increased transverse diameter, degeneration, and hypertrophy predominantly in right ventricular cardiomyocytes. The authors concluded that while hyperthyroid cats exhibit cardiac hypertrophy, it is less prominent and affects the heart more diffusely. Therefore, although hyperthyroidism is considered a differential diagnosis, the hypertrophic pattern observed in HCM differs distinctly. Therefore, the macroscopic patterns of hypertrophy associated with HCM and hyperthyroidism are expected to differ, although additional studies are required to confirm this.

It is also important to highlight, as mentioned earlier, the possibility of transient myocardial thickening, which tends to resolve over time and is generally associated with a preceding event. In such cases, accurate diagnosis is crucial, as there are specific criteria that must be evaluated before a cat can be classified as having transient thickening. The diagnostic criteria for this condition are based on the presence of initial wall thickening associated with CHF, normalization of LVwall thickness, atrial diameter, and cardiac function in follow-up examinations, resolution of CHF, and discontinuation of diuretic therapy. These findings were described by Matos et al. (24) and Romito et al. (25), who also reported associations with prior stress-related events such as anesthesia and road traffic accidents. In the study by Matos et al. (24), among 21 cats with transient thickening, 15 had a history of procedures involving anesthesia, vaccinations, or pain. Similarly, Romito et al. (25) found antecendents factors (e.g., anesthesia, infectious diseases, castration) in 14 of the 27 evaluated cats. Both authors emphasized that the outcomes were favorable, with a marked decrease in myocardial thickness, improvement in echocardiographic parameters, and reduced need for pharmacological therapy.

### 3.2 Laboratory evaluation, biomarkers, and other diagnostic options

Laboratory evaluation in feline HCM is non-specific, as no hematologic or biochemical abnormalities are pathognomonic for HCM phenotype (27). Nonetheless, laboratory testing remains valuable for assessing the patient's overall health status, which can help detect comorbidities. It may also raise suspicion of underlying infectious or zoonotic diseases, although more specific tests are needed to confirm these conditions. In feline patients, testing for the two most common retroviruses, feline leukemia virus and feline immunodeficiency virus, is crucial. Retrovirus coinfection can exacerbate clinical signs and complicate the disease course through immunosuppression, although further investigation is needed to clarify these effects (60). Laboratory tests also facilitate diagnosis or exclusion of underlying diseases such as CKD and hyperthyroidism (6, 53). Genetic testing, performed from blood or oral swabs, can support screening in breeding animals, young cats, or individuals from affected lineages (18, 20, 21, 61). However, a negative result does not preclude future development of HCM, and continued clinical monitoring remains necessary (18, 20, 21, 61). Thus, regular clinical follow-up remains essential, even in cats testing negative for HCM-associated mutations.

From a hematological perspective, few parameters have demonstrated clinical utility in feline HCM. Stanzani et al. (62) reported that red cell distribution width (RDW) was significantly higher in cats with HCM and CHF compared to controls, and also differed between cats with and without CHF. Cats with RDW values below 19.1 had longer median survival, suggesting its potential prognostic relevance (62). Other potential and valuable option is the neutrophil-to-lymphocyte ratio (NLR). The NLR has also been proposed as a prognostic indicator (63). Cats at stage C showed higher NLR values than healthy or stage B cats, with further increases in those exhibiting spontaneous echocardiographic contrast or atrial thrombi (63). Elevated NLR was associated with both HCM and CHF and with shorter survival, supporting its potential as a prognostic biomarker (63). To date, few studies have compared laboratory parameters between cats with HCM and healthy controls, likely due to comorbidities complicating interpretation of standard tests.

Platelet function has been increasingly investigated in cats with the HCM phenotype due to the risk of thrombus formation. Since 2008, hypercoagulability in cats with HCM has been under study. Jandrey et al. (64), using a platelet function analyzer, reported that closure time was 64 s in healthy cats and 74 s in cats with HCM, although the difference was not statistically significant, highlighting the need for further investigation. More recently, Algan et al. (65) evaluated the utility of platelet large cell count and its ratio in cats with HCM. They observed higher counts in cats with HCM, reduced values in those with ATE, and a gradual increase from stage A (control) to C/D (symptomatic). The discriminative ability was higher for platelet large cell count, while the ratio served as an auxiliary parameter, contributing to the assessment of disease severity and thrombotic risk in HCM cats. Additionally, Yoon and Li (66) investigated the NLR and platelet:neutrophil ratio (PNR) in cats with HCM and ATE. Cats with ATE had higher NLR compared to those with HCM or healthy cats, while PNR was lower in cats with ATE. Clinically affected cats also had higher NLR than subclinical cases, and cats with NLR below 40 exhibited shorter median survival, indicating its potential as a predictor of mortality. These findings suggest that laboratory indices involving platelets may provide useful auxiliary information for diagnosis, monitoring, and thrombotic risk assessment in cats with HCM.

**Figure 2:**
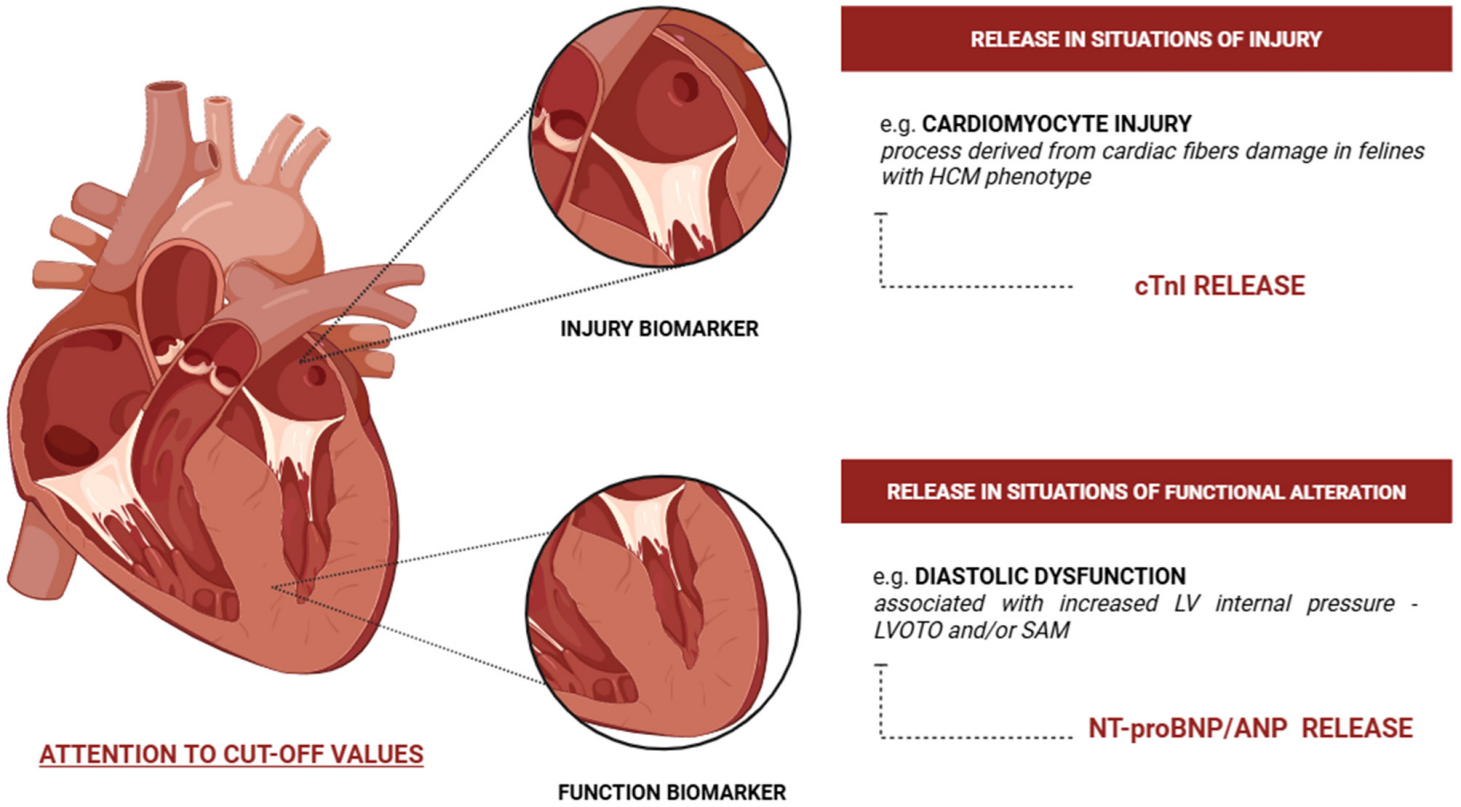
Characterization of the release of cardiac biomarkers of function and injury in situations such as atrial dilation and volume overload. Created by the authors with BioRender.com.

Biomarkers are most useful as adjuncts to routine laboratory testing, providing additional information on cardiac function, myocardial injury, disease progression, and serving as prognostic indicators (4, 6, 18, 67–69). However, limitations such as availability, cost, and turnaround time may restrict their clinical use (2, 6, 40, 67). In Brazil, biomarker use in routine practice remains limited due to high costs, and echocardiography is more available. Moreover, biomarkers indicate structural changes, whereas echocardiography remains a more specific and cost-effective diagnostic tool. Cardiac biomarkers include atrial natriuretic peptide (ANP), N-terminal pro-atrial natriuretic peptide (NT-proANP), B-type natriuretic peptide (BNP), NT-proBNP, and cTnI (Figure 2; Table 1) (6, 30, 67, 69, 70). NT-proANP and NT-proBNP are markers of cardiac function, whereas cTnI reflects myocardial injury (27). Elevation of functional biomarkers, particularly NT-proBNP, is primarily associated with volume overload and cardiomyocyte stretch (67, 69), while injury biomarkers such as cTnI increase following direct myocardial damage (71) (Figure 2).

**Table 1:** Feasibility of biomarkers in felines with the HCM phenotype, including potential options.

**Feasibility of using NT-proBNP for the HCM phenotype according to Fries et al**. **(****15****)**			
**Clinical indication**	**NT-proBNP**	**SNAP**	**Conclusions**
Feline with respiratory symptoms possibly associated with congestive heart failure (CHF)	< 100	Normal	CHF unlikely
	100–270	Abnormal	Possible CHF; additional tests or therapeutic trials should be performed
	270		CHF highly likely
Asymptomatic feline with cardiac risk factors (e.g., murmur, arrhythmia, gallop rhythm)	< 100	Normal	Heart disease should not be considered
15.6-2.2,-1.3498pt	>100	Abnormal	Heart disease highly likely; confirmatory testing (e.g., echocardiogram) is recommended
**Feasibility of using cTnI, ANP, and NT-proANP for the HCM phenotype**			
cTnI	• Myocardial injury marker • Predictor of mortality (0.7 ng/ml) • Plasma concentrations of 0.163 ng/ml (sensitivity 62% and specificity 100% – differentiates healthy and asymptomatic HCM without left atrial dilation) • Cutoff value 0.18 ng/ml: sensitivity 60.7% and specificity 82.4% for differentiating healthy from diseased cats. • 0.234 ng/ml identifies severe stage (sensitivity 95.0%; specificity 77.8%) • cTnI is associated with HCM, SAM, left ventricular wall thickness, and LA/Ao ratio • Values may vary by breed and sex in healthy animals		
ANP NT-proANP	• Elevated values in cats with heart disease, CHF, and left atrial dilation • ANP >77.5 pg/ml (sensitivity 66.3%; specificity 84.6%) – distinguishing cats with cardiomyopathy from healthy individuals • Left atrial dilation – ANP >110.9 pg/ml (sensitivity 73.6%; specificity 93.5%) • Association between NT-proBNP, atrial size, and left ventricular free wall thickness • NT-proANP is not valid for differentiating healthy and cardiomyopathy cats		
**New potential biomarkers in HCM phenotype**			
Potential biomarkers for complementary diagnostic, monitoring, or prognostic use in cats with HCM include those related to vasoconstriction (e.g., endothelin), remodeling (e.g., LOX, TGF), inflammation and immunomodulation (e.g., IL-18, galectin-3, NETs), thrombosis and hemostasis (e.g., D-dimer, prothrombin time), cellular communication and myocardial structure (e.g., connexin 43, FBN2), as well as regulatory genes and proteins (e.g., ITGAM, ELOB, ZNF316, ENTPD8, WNT5A). However, further clarification is still needed, particularly from a clinical and practical perspective			

Information available from the studies (37, 40, 70, 72–92).

Biondo et al. (72) investigated whether the distribution of ANP and BNP is altered in feline HCM. In healthy cats, immunoreactivity for both peptides was restricted to the atria, predominantly in cardiomyocytes adjacent to the endocardium, while BNP was also detected in nerve fibers and small intracardiac capillaries (72). In cats with HCM, atrial staining for ANP and BNP became more diffuse and less sharply defined, and mild BNP staining appeared in ventricular cardiomyocytes (72). These findings indicate that HCM alters the normal atrial-restricted distribution of natriuretic peptides, consistent with structural and functional remodeling. Recently, Heishima et al. (81) evaluated ANP concentrations in healthy and cardiomyopathic cats and assessed their diagnostic performance. ANP levels were significantly higher in cardiomyopathic cats with CHF and left atrial dilation, whereas values in cats without atrial dilation did not differ from controls. An ANP cutoff of 77.5 pg/ml differentiated cardiomyopathy from health with 66.3% sensitivity and 84.6% specificity, while a cutoff of 110.9 pg/ml identified cats with atrial dilation with 73.6% sensitivity and 93.5% specificity (81). These results suggest that ANP increases primarily in more advanced disease stages, providing supportive, but not definitive, diagnostic value (Table 1). However, their sensitivity and specificity values do not categorize them as the most precise diagnostic options, although they provide valuable insights into the disease (Table 1). MacLean et al. (75) evaluated NT-proANP immunoreactivity in healthy and cardiomyopathic cats and observed higher concentrations in diseased animals; however, the differences were not statistically significant, limiting its diagnostic utility. In contrast, Parzeniecka-Jaworska et al. (78) reported higher proANP levels in Maine Coon cats with HCM, with moderate correlations with age (*r* = 0.56), ejection fraction (*r* = 0.53), and left ventricular systolic diameter (*r* = 0.48). These findings suggest that, while NT-proANP has limited diagnostic value, proANP may function as a supportive biomarker associated with structural and functional disease severity (Table 1).

Since 2009, NT-proBNP has been investigated in feline HCM, initially evaluated by Hsu et al. (76) in purebred Maine Coon cats and their crossbreeds to assess its potential as a screening biomarker. They found that NT-proBNP concentrations were higher in cats with severe disease, suggesting predictive value in advanced stages. However, the peptide failed to reliably identify mild or moderate HCM. Importantly, cats carrying the A31P mutation in the MYBPC3 gene also showed higher NT-proBNP concentrations, indicating that genetic status may influence biomarker release (76). Overall, the authors concluded that NT-proBNP was not a reliable diagnostic marker for early or intermediate disease. Similar findings were later described by Singh et al. (93), who also reported poor performance of NT-proBNP in early HCM (Table 1). MacLean et al. (75) observed a moderate correlation with left ventricular free wall thickness (*r* = 0.42) and a weak correlation with left atrial dimension (*r* = 0.35), with multivariate analysis showing a linear association between NT-proBNP, atrial size, and ventricular free wall thickness. Wess et al. (77), employing a sample size roughly five times larger than prior studies, found significantly higher NT-proBNP levels in affected cats, particularly in severe cases. They proposed diagnostic thresholds of >49 pmol/L (sensitivity 97.8%, specificity 66.7%), >100 pmol/L (sensitivity 92.4%, specificity 93.9%), and >150 pmol/L (sensitivity 88%, specificity 100%), recommending >100 pmol/L for mild disease. Fox et al. (94) confirmed NT-proBNP's ability to distinguish healthy cats from those with occult cardiomyopathy, while Abdelhaleem et al. (92) reported that a cutoff of 213 pmol/L achieved 72% sensitivity and 88% specificity for differentiating primary from secondary cardiomyopathies. Overall, NT-proBNP reliably differentiates healthy cats from those with HCM, including early-stage disease, although its performance may be affected by genetic factors such as the A31P mutation.

Currently, NT-proBNP is considered one of the most widely used cardiac biomarkers, supported by its stability, long half-life, and consistent clinical application (18, 71, 95–100). It is important to note that NT-proBNP concentrations can be influenced by non-cardiac conditions (e.g., renal disease, neoplasia, inflammatory states) (98). NT-proBNP is useful in HCM but should not be interpreted in isolation. In the presence of systemic comorbidities, particularly inflammatory or renal disorders, elevated values may not exclusively reflect cardiac dysfunction. Cats with lower NT-proBNP concentrations show higher survival rates (101). Despite this, its use in healthy cats is not recommended due to high individual variability (79), although it remains reliable in severe HCM (40). For occult disease, point-of-care (POC) ELISA testing is an available option. Machen et al. (70) demonstrated that POC testing can detect moderate to severe forms of occult HCM, but not mild disease, emphasizing that echocardiography remains essential. According to Harris et al. (80), a positive POC result strongly supports the presence of cardiomyopathy, while a negative result does not reliably exclude it. A key advantage of NT-proBNP is its ability to differentiate cardiac from respiratory causes of dyspnea (97). Hezzell et al. (97) reported sensitivity of 95.2% and specificity of 87.5% for this purpose. Elevated NT-proBNP concentrations are also associated with increased risk of cardiac events in cats with left atrial enlargement (99), consistent with findings from van Hoek et al. (83) and Hanås et al. (103). Similar associations have been described in cats with systolic anterior motion (SAM) and atrial dilation (101, 102, 104, 105). Overall, NT-proBNP remains a valuable biomarker in cats with HCM, supporting diagnosis, assessment of dyspnea etiology, and prognostic evaluation, particularly in cases with structural cardiac changes. It is also worth highlighting the usefulness of these tests (e.g., POC ELISA NT-proBNP) during pre-anesthetic evaluations prior to surgical procedures, especially in situations where conventional echocardiography is not available or the animal's guardian is unable to cover all the costs. In this context, given their beneficial effects, these tests can provide additional information.

Another biomarker used to assess myocardial injury is cTnI. Its utility in cats with HCM has been recognized since 2002, when Herndon et al. (106) observed elevated concentrations in cardiomyopathy patients, with high sensitivity and specificity for moderate to severe disease, and further increases in cats with CHF. Myocardial injury appeared progressive and associated with fibrosis. Hertzsch et al. (107) later demonstrated diagnostic potential even in clinically healthy cats, using a cutoff of >0.06 ng/ml (sensitivity 91.7%, specificity 95.4%). Because endocrine disorders may influence cTnI, Sangster et al. (58) proposed that hyperthyroid cats can show elevated values, a finding confirmed by Janus et al. (54), who reported myocardial changes secondary to hormonal disease. Elevated cTnI is also linked to worse outcomes (108). Borgeat et al. (67) reported that concentrations above 0.7 ng/ml predict mortality in HCM, even in the absence of CHF or left atrial dilation. The multicenter study by Hori et al. (82) showed higher cTnI in cardiomyopathy cats than in controls, and in CHF patients compared with asymptomatic cats. A cutoff of 0.163 ng/ml achieved 62% sensitivity and 100% specificity for subclinical HCM, although false negatives limited its screening utility. Values above 0.234 ng/ml were associated with cardiac dysfunction (sensitivity 95.0%, specificity 77.8%). Similar findings were reported by van Hoek et al. (83) and Hertzsch et al. (107), and in cats with SAM (104, 105). Hanås et al. (109) also showed that cTnI values vary with breed and sex in healthy cats, and in HCM patients they correlated with left ventricular wall thickness and LA/Ao ratio, with higher values in those with left atrial dilation.

It is worth mentioning that cTnI concentrations may be altered in cases of transient myocardial thickening, with some cats showing markedly elevated values compared to the upper reference limits. In the study by Matos et al. (24), among 21 cats diagnosed with transient myocardial thickening, cTnI was measured in 13, showing median values of 2.1 ng/ml (range: 0.05–63.8 ng/ml), which further supports that cTnI concentrations may also increase in cases of transient myocardial thickening, reflecting reversible myocardial injury. Another utility of cTnI is its applicability during pre-anesthetic laboratory evaluations, especially when the availability of conventional echocardiography is limited or when financial constraints make it impossible to perform the procedure. Cardiac troponin I (cTnI) may also provide valuable information for assessing therapeutic efficacy and monitoring in cats with the HCM phenotype, although further studies are needed to rigorously evaluate its changes in patients with LVOTO. Satomi et al. (110) demonstrated that cTnI can be elevated in cats with LVOTO, suggesting a potential association between obstruction and myocardial injury due to worsening of the condition. The authors also highlight that cTnI may be used as a monitoring parameter in LVOTO cases, but additional investigations are required. In turn, Jackson et al. (111) evaluated the effect of atenolol in cats with subclinical HCM, showing that the drug improves LVOTO, indicating that cTnI could be a valid diagnostic and monitoring marker in these cases.

More recent studies confirmed that cTnI increases with advanced HCM, particularly obstructive forms, correlating with LVOT tract velocity and NT-proBNP (8, 92). A cutoff of 0.18 ng/ml differentiates healthy from affected cats with sensitivity around 61% and specificity near 83%. Although ANP, NT-proANP, BNP, NT-proBNP, and cTnI provide valuable adjunctive information, their variability and limited diagnostic performance highlight the continued importance of imaging, particularly echocardiography, for confirming structural cardiac disease in cats with an HCM phenotype. In a study by Shimoda et al. (9), 26 cats with cardiomyopathy were classified as with or without CHF based on the presence of pulmonary edema or pleural effusion. The diagnostic performance of echocardiographic indices and circulating biomarkers was assessed, revealing that the LA/Ao ratio exhibited the highest accuracy (AUC 0.952), followed by ANP (0.915), cTnI (0.861), and NT-proBNP (0.830) (9). Notably, combining biomarkers with the LA/Ao ratio did not improve discrimination. These findings indicate that the LA/Ao ratio is the most reliable parameter for identifying cats with CHF, outperforming both individual biomarkers and their combined use, and reinforcing its central role in the clinical evaluation of cardiomyopathy.

Experimental biomarkers have been explored in HCM, targeting fibrosis, myocardial remodeling, immune modulation, coagulation, and intercellular communication (Table 1). Endothelin, a potent vasoconstrictor peptide, was elevated in cats with cardiomyopathy, particularly in those with atrial septal defects (74), though its diagnostic utility remains uncertain. Coagulation markers such as D-dimer and prothrombin time were evaluated by Jiwaganont et al. (89), who reported increased D-dimer and shortened prothrombin time in symptomatic HCM cats. The same study identified differential expression of genes including integrin subunit alpha M (ITGAM), fibrillin-2 (FBN2), elongin B (ELOB), zinc finger protein 316 (ZNF316), and ectonucleoside triphosphate diphosphohydrolase 8 (ENTPD8), suggesting their involvement in HCM pathophysiology. Fibrosis-related biomarkers were investigated by Cheng et al. (86), who documented myocardial upregulation of lumican, lysyl oxidase (LOX) isoenzymes, and transforming growth factor beta (TGF-β) isoforms, similar to findings in human HCM. Galectin-3 (Gal-3), a member of the galactoside-binding lectin family, has emerged as a potential fibrosis biomarker (112). Stack et al. (87) and Demeekul et al. (90) reported increased circulating Gal-3 concentrations in cats with HCM, although correlations with echocardiographic indices varied, highlighting the need for further validation.

Immunomodulatory biomarkers, including interleukin-18 (IL-18), insulin-like growth factor binding protein-2 (IGFBP-2), and WNT family member 5A (WNT5A), were examined by Chong et al. (88). IGFBP-2 and WNT5A were elevated in subclinical disease, whereas IL-18 and WNT5A increases were more pronounced in cats with congestive heart failure (CHF). Prothrombotic mechanisms have also been explored: neutrophil extracellular traps (NETs) were identified in intracardiac thrombi (85), and circulating cell-free DNA (cfDNA) and reduced citrullinated histone H3 (citH3) correlated with atrial function in cats with HCM and SAM of the mitral valve (84, 85). Platelet procoagulant responses and altered surface markers in HCM were further described by Shaverdian et al. (37). Additional findings include reduced myocardial expression of connexin-43 (Cx43) in fibrotic regions (91), suggesting impaired electrical coupling. Increased circulating insulin-like growth factor 1 (IGF-1) and 3-methyl-histidine (3-MH) have also been reported, reflecting metabolic alterations (113). Multiple genes and microRNAs associated with hypoxia-inducible factor 1-alpha (HIF1α), TGF-β signaling, hypertrophy, and immune activation were implicated in a transcriptomic analysis by Joshua et al. (114). Collectively, these biomarkers enhance understanding of molecular and inflammatory pathways in HCM and may inform future therapies.

In general, biomarkers investigated in feline HCM span multiple biological pathways, including fibrosis, myocardial remodeling, immune modulation, coagulation, and intercellular communication. These studies broaden understanding of disease mechanisms and highlight parallels with human cardiomyopathy, suggesting potential contributions to disease onset, progression, and complications such as arterial thromboembolism (ATE) or CHF. Despite these advances, their clinical applicability remains limited, as most have been evaluated only in experimental settings. Their predictive, diagnostic, and prognostic utilities are still unclear, and reproducibility in real-world veterinary practice is uncertain. At present, these biomarkers should be viewed as research tools that enrich mechanistic insight rather than clinically actionable parameters. Future studies should aim to better define and validate the relevance of these experimental biomarkers and clarify whether they may eventually support diagnostic or therapeutic decision-making. Until such evidence exists, their practical utility remains uncertain, and clinical interpretation should be approached with caution.

### 3.3 Cardiovascular diagnostic methods

Cardiac diagnostic evaluation in HCM encompasses examinations that assess thoracic cavity structures (radiography), cardiac electrical conduction (electrocardiography), and cardiac structure and function (echocardiography). Ideally, these diagnostic tools should be employed complementarily, as their sensitivity and specificity vary. Among them, echocardiography is considered the gold standard for feline HCM diagnosis due to its capacity for detailed structural and functional assessment (2, 18). Moreover, these examinations are indispensable for routine cardiovascular monitoring, especially in felines diagnosed with or predisposed to HCM. Regular monitoring provides a comprehensive evaluation of clinical status, informs therapeutic adjustments, facilitates detection of regional and systemic complications (2, 18). Furthermore, it enables tracking of progressive thickening of the LV free wall and/or interventricular septum (2, 18).

#### 3.3.1 Radiographic evaluation

Felines with the HCM phenotype are more likely to develop pulmonary edema than pleural effusion (5, 95). Pulmonary edema is more strongly associated with dyspnea, although pleural effusion can also contribute to respiratory distress (5, 95). Thoracic radiographs readily reveal these changes, making radiography a valuable tool for evaluating respiratory alterations in the clinical context (6). A minimum of two projections, latero-lateral and ventrodorsal, is recommended for a reliable assessment (115). Radiographic examinations should be performed only in stabilized patients, as restraint during the procedure may trigger decompensation (5, 116).

Cats with pleural effusion may initially show minimal dyspnea, as compensatory mechanisms maintain adequate hemoglobin oxygen saturation (117). When PaO_2_ falls below 60 mmHg, oxygen delivery becomes critically impaired, and acute increases in demand, manifested as orthopnea, thoracic limb abduction, neck extension, or open-mouth breathing, can trigger decompensation (117). Pleural effusion may also cause dyspnea through hypoventilation and lung atelectasis, potentially leading to death, underscoring the need to stabilize patients before radiography (117). Careful clinical assessment during triage is essential, as stress-induced tachycardia may occur (118). In cats with HCM, reduced LV cavity diameter due to hypertrophy limits stroke volume, predisposing to circulatory failure, which may be worsened by SAM (18, 28). The findings highlight that, although radiography is useful for auxiliary diagnosis, it should only be performed after the patient is stabilized. Performing radiography in unstable patients can trigger decompensation (e.g., due to manual restraint), which may be fatal.

Compared to echocardiography, radiographic assessment has relatively low sensitivity and specificity for diagnosing HCM, which imposes inherent limitations (119, 120). Thoracic radiography demonstrates an overall diagnostic accuracy of approximately 70% in evaluating the HCM phenotype, and automated image analysis may further enhance its performance (119). The variability of radiographic findings reflects both the heterogeneity of the disease and differences in severity (115). As Pellegrino (121) notes, “thoracic radiograph findings depend on the extent of hypertrophy, degree of myocardial dysfunction, presence of chamber enlargement, and severity of circulatory congestion.” Beyond visual inspection, several radiographic assessments are employed to evaluate cardiac size and morphology, including the clock-face analogy, Vertebral Left Atrial Size (VLAS), Vertebral Heart Score (VHS), and Heart Size Vertebral Ratio (HSVR) (120, 122–124). While these measures are useful, their limitations and appropriate clinical applications must be clearly defined and critically assessed to provide valid diagnostic support for the HCM phenotype.

The clock-face analogy overlays a schematic onto the cardiac silhouette on radiographs to localize chamber enlargement (124). In this model, the 11:00–2:00 segment represents the left atrium (LA), 2:00–5:00 the left ventricle (LV), 5:00–8:00 the right ventricle (RV), 8:00–9:00 the right atrium (RA), and 9:00–12:00 the great vessels (124). Enlargement of the 11:00–2:00 segment suggests LA dilation, but in cats, age-related cardiac positioning against the sternum may limit its accuracy (124). An alternative is the VLAS, which measures a line from the ventral carina to the caudal LA margin intersecting the dorsal caudal vena cava, expressed in vertebral body units (120, 123). While VLAS has been applied in cats, no significant differences were found between HCM and CHF cases, highlighting the need for further studies to determine its diagnostic utility.

A modified VHS technique, termed LA-VHS, was proposed by Schober et al. (122). It measures a line from the heart base to the apex and a second line from the caudal dorsal edge of the LA to the dorsal margin of the caudal vena cava, both expressed in vertebral units (122). This method demonstrated high specificity but limited sensitivity for detecting LA enlargement in cats. The distinction between VLAS and LA-VHS lies in their measurement planes: VLAS reflects the transverse dimension, whereas VHS involves both the long (base-to-apex) and short (right-to-left atrium) cardiac axes (126). These axes are transposed onto the vertebral column starting at the fourth thoracic vertebra, with VHS calculated as their sum in vertebral units, rounded to the nearest 0.1 vertebra. Menoyo (124) and Litster et al. (127) reported that the mean VHS in domestic shorthair cats is 7.5 ± 0.3–0.5 vertebrae, with an upper normal limit of eight vertebrae; however, breed and thoracic conformation can influence these values (128, 129). A major limitation of VHS in cats is the scarcity of systematic studies establishing normative cardiac size parameters and diagnostic accuracy in diseased felines (130). Although thoracic radiography is presumed to have high specificity, its accuracy declines in cats with cardiomyopathy. A newer method, the HSVR, calculates the sum of the heart's longitudinal and transverse axes as a dimensionless index. HSVR has shown concordance with VHS and may serve as a useful alternative for feline cardiac assessment (125).

Given the potential atrial involvement secondary to the pathophysiological mechanisms of the HCM phenotype, thoracic radiographs are expected to demonstrate LA enlargement. Subjective assessment of this alteration may be assisted by the clock-face analogy (120, 124), and generalized cardiomegaly may also be apparent (115, 122). In a subset of cases, cats exhibit the ‘Valentine Heart' shape, defined by biatrial expansion producing widening at the cardiac base with tapering of the apex (131, 132). Winter et al. (131) found that this shape corresponded to biatrial enlargement in 41% of cats with cardiomyopathy, but only 8% of cats with confirmed HCM, a finding also noted by Kim et al. (120). Although the pattern has low sensitivity for HCM, it remains a relevant radiographic marker of cardiomyopathy.

Diana et al. (132) characterized radiographic abnormalities in cats with cardiomyopathy and pulmonary edema. Of 103 cats reviewed, 71 met inclusion criteria, and 64.8 % (46/71) exhibited an HCM phenotype. Radiographs of affected cats revealed cardiac changes, including subjective cardiomegaly, caudal contour indentations, the “Valentine Heart” shape, and a double-wall appearance (superimposition). Vascular alterations were also observed, such as pulmonary arterial dilation and tortuosity with an artery-to-vein ratio close to 1:1. Parenchymal abnormalities included interstitial and mixed infiltrates with multifocal, symmetrical ventrocranial and ventrocaudal distribution, accompanied by bronchial patterns. Additional findings included pleural effusion, caudal vena cava dilation, and aerophagia. Collectively, these alterations highlight the wide variability of radiographic manifestations in feline HCM with respiratory compromise.

Kim et al. (120) evaluated thoracic radiography for detecting CHF in cats with the HCM phenotype. Seventy-eight cats were included, 43 with HCM (21 with CHF) and 35 controls. Mean VHS was higher in affected cats (8.57 ± 0.4) than controls (7.57 ± 0.46). No significant difference was observed between HCM cats with and without CHF. For CHF detection, VHS showed moderate sensitivity (76.19%) and low specificity (40.91%) (120). Radiographic signs of LA enlargement, such as carinal elevation and auricular or LA bulging, were more frequent in HCM cats. Auricular bulging correlated with the echocardiographic LA/Ao ratio. LA dilation had high specificity (94.12%) but low sensitivity (17.5%). CHF secondary to HCM was also associated with increased LA diameter and pulmonary vein dilation (120). These findings support radiography as an ancillary tool in cats with HCM. While it cannot distinguish cardiomyopathy subtypes, it is useful for diagnosing and monitoring CHF, detecting pulmonary edema, and excluding differential diagnoses. Further studies are needed to validate and refine its diagnostic value (120).

#### 3.3.2 Electrocardiographic evaluation

Electrocardiographic evaluation of cats exhibiting the HCM phenotype constitutes a valuable tool for assessing cardiac electrical conduction. Based on the underlying pathophysiology, electrical conduction abnormalities are anticipated (5, 6, 16, 133–136). Although electrocardiography represents a valid diagnostic modality, its sensitivity and specificity for detecting cardiac diseases, particularly HCM, are limited (30). In this context, electrocardiography is primarily useful for raising initial suspicion of conduction abnormalities. No pathognomonic or definitive electrocardiographic morphological alterations exist that conclusively confirm the presence of the HCM phenotype (30). Affected cats may present with various arrhythmias, necessitating further evaluation through echocardiography for definitive diagnosis. Commonly reported arrhythmias and conduction abnormalities include supraventricular and ventricular tachyarrhythmias, ventricular pre-excitation, QRS complex widening, electrical axis deviations, increased R wave amplitude and atrial fibrillation (5, 6, 16, 133–136). Additional electrical findings may include bundle branch blocks, atrioventricular blocks and left fascicular blocks (134, 137). Depolarization and repolarization abnormalities are less frequent, although some cats may demonstrate transient ST-segment elevation (138, 139). The recent study by Szlosek et al. (140) demonstrated that the most frequently observed arrhythmia in cats is the presence of ventricular premature complexes. Male and older cats (10–13 years) showed a higher likelihood of developing arrhythmic events. It is important to emphasize that the presence of arrhythmias is not, by itself, indicative of cardiac disease, as they may also occur in cases of electrolyte disturbances, neoplasms, or other comorbidities. Moreover, not all arrhythmias require pharmacological intervention, as the need for treatment should be determined according to the nature and severity of the arrhythmia observed.

More detailed investigations into electrical abnormalities have concentrated on ventricular arrhythmias, offering potential screening tools for HCM. Bastos et al. (138) assessed the Tpeak-Tend (Tpte) interval, defined as the duration from the peak to the end of the T wave, and its ratio relative to the QT interval. They reported that cats with HCM exhibited increased Tpte values in leads DII, aVR, aVL, and aVF. Notably, a Tpte value exceeding 27.5 ms in lead aVF demonstrated 83.3% accuracy for detecting HCM. Felines are considered predisposed to HCM if the Tpte surpasses 27.5 ms in leads aVR and aVF, and 26.5 ms in lead aVL. These authors concluded that the Tpte interval may serve as a valuable parameter in HCM screening. In a subsequent study, Bastos et al. (141) observed that cats with HCM exhibited prolongation of QT and QRS intervals, alongside increased ventricular electrical instability. These findings suggest that both the QT and corrected QT intervals could be useful parameters in HCM investigation. Despite the low diagnostic accuracy of ECG for HCM, it remains a valuable tool for ongoing monitoring. Ideally, 24-h Holter monitoring should be employed to enable extended electrical tracing acquisition and facilitate arrhythmia detection in cats with HCM phenotype (142, 143). Nonetheless, as Sousa et al. (27) noted, despite its clinical advantages, Holter monitoring is less frequently used in Brazilian practice due to barriers such as high equipment costs, limited availability of trained personnel for interpretation, and financial constraints faced by pet owners.

#### 3.3.3 Echocardiography evaluation

Echocardiography is regarded as the imaging modality with the highest diagnostic accuracy for identifying the HCM phenotype in cats (2, 6, 40, 45, 71). It enables comprehensive assessment of both cardiac structure and function in accordance with criteria established by the American College of Veterinary Internal Medicine (ACVIM) (2). Accurate echocardiographic evaluation requires operator expertise to ensure diagnostic reliability (9, 30, 40). Häggström et al. (30) and Luis Fuentes and Wilkie (40) emphasize that the examination can be performed and assessed using M-mode, two-dimensional (2D), color Doppler, pulsed Doppler, or a combination thereof. For the HCM phenotype, echocardiographic findings may include left ventricular hypertrophy with or without LA dilation, atrioventricular valve regurgitation, SAM, atrial thrombi, and spontaneous echocardiographic contrast (Table 2) (5, 18, 28). Diagnosis can be challenging due to the asymmetric distribution of myocardial hypertrophy and interference from papillary muscles, which may affect measurement accuracy (2, 18, 144). Reference values are method-dependent, and measurements obtained via M-mode and 2D imaging are not interchangeable (2).

**Table 2:** Classification according to Luis Fuentes et al. (2) for left ventricular free wall and/or interventricular septal myocardial thickness, as well as the most frequent echocardiographic findings in cats with the HCM phenotype.

**Classification of the thickness of the LV free wall and/or interventricular septum for HCM**	
< 5 mm	Healthy feline with no cardiac hypertrophy
5–6 mm	Feline in a borderline range or under evaluation The animal needs to be reassessed for a more accurate analysis
≥6 mm	Feline shows cardiac hypertrophy with strong evidence of the hypertrophic phenotype
**Echocardiographic features for classification of the HCM**	
**phenotype**	
16.5-1,16.5**Parameter**	**Description**
LV hypertrophy LV heterogeneity	Concentric hypertrophy–asymmetric (most common) or symmetric *Note: Do not include the papillary muscles in the measurement*
LV internal diameter and volume	Reduction in end-diastolic diameter and stroke volume due to the presence of hypertrophy–resulting in impaired ventricular ejection
LA dilation	Enlargement of the left atrium (LA) due to volumetric remodeling–measured by an LA/Ao ratio greater than 1.5
Systolic anterior motion (SAM)	Mitral leaflet displacement (septal) into the left ventricular outflow tract–associated with elevated ventricular pressure
Dynamic obstruction of the LV outflow tract	Condition characterized by obstruction of the ventricular outflow tract to the aorta–associated with asymmetric hypertrophy (more cranial portion of the interventricular septum) and SAM–resulting in a ‘scimitar-shaped' flow (observed in spectral doppler). The presence of one or both leads to LVOTO–increased pressure and worsening of the clinical condition
LV hypokinesia	Reduced and abnormal movement of the LV–associated with functional impairment
Thinning of the left ventricular free wall	They may present thinning of the LV, especially the free wall–a more severe phenotype associated with shortened survival time
Ejection fraction (EF%) e fractional area change (FAC)	Decrease in atrial EF% and FAC
Alterations in systolic excursions of the annular plane	Decreased septal and free wall MAPSE. Decreased TAPSE
Transmitral flow	Alteration in the E and A wave size pattern (>1.77 predict CHF)
S' wave	Reduction in S' values
Strain	Changes in myocardial deformation and velocity such as longitudinal and circumferential planes
Mitral leaflet	Increased length of the septal leaflet to the interventricular septum
Aorto-septal angle	Could be associated with the presence of a murmur and cranial septal hypertrophy, but further studies are needed

Information available in the studies (2, 17–19, 29, 31, 40, 46, 95, 114, 143–157, 159).

M-mode echocardiography is commonly used for measuring interventricular septal and left ventricular free wall thickness but may yield unreliable results in cases of asymmetric hypertrophy (2, 144). Therefore, it is recommended to acquire measurements using 2D imaging in both short- and long-axis views (2). At least two measurements should be taken for each region of interest (ROI), for example, longitudinal and papillary axis, by the interventricular septum and the left ventricular free wall in diastole (2, 30). Papillary muscles should be excluded from measurements to avoid overestimation of myocardial thickness (2, 30). Use of a single echocardiographic technique does not preclude the need for complementary diagnostic methods. Moreover, diagnosis should not rely solely on septal or ventricular wall thickness but also consider additional echocardiographic parameters to suggest the HCM phenotype (Table 2) (18), especially when reversible or transient myocardial hypertrophy is suspected (158). Genotypic variation influences echocardiographic findings in cats with HCM. In this context, Stern et al. (159) demonstrated that homozygous cats can exhibit more pronounced myocardial thickening and higher LA/Ao ratio compared to heterozygous individuals. LV outflow tract obstruction (LVOTO) due to SAM, with associated regurgitant flow in the left ventricular outflow tract (LVOT), is frequently observed, particularly in cats carrying the A31P mutation (Table 2) (159). A frequently reported finding in the LVOT region is septal hypertrophy, which is regarded as a predilection site for HCM (2).

Echocardiography facilitates detailed evaluation of cardiac structure and function, assessment of hypertrophy distribution, detection of diastolic dysfunction, transvalvular flow analysis, identification of obstructive patterns (LVOTO/SAM), thrombi, spontaneous contrast, mitral annular plane systolic excursion (MAPSE), and color and tissue Doppler imaging (Table 2). It plays a crucial role in disease staging and longitudinal monitoring (Table 2). According to Luis Fuentes et al. (2), myocardial wall thickness below 5 mm is considered normal. Myocardial thicknesses between 5 and 6 mm fall into a diagnostic “gray zone,” requiring reevaluation as recommended by the 2020 consensus. Hypertrophy is confirmed with myocardial thickness ≥6 mm, constituting a key diagnostic criterion for HCM, excluding false tendons or trabeculations (Table 2) (2). It is also important to mention, in addition to the general challenges of performing echocardiographic examinations in cats, the frequent occurrence of stress-induced tachycardia. Cats subjected to manual restraint for complementary examinations often experience a high level of stress, which negatively affects the accuracy of cardiovascular assessment. Stress-related tachycardia can alter flow patterns, particularly those involving the left ventricular outflow tract in cats with LVOTO and/or fusion of transmitral or tissue Doppler (e′/a′) waves, thus preventing accurate evaluation of diastolic function, especially by inexperienced operators. Consequently, overestimation or misinterpretation may occur, compromising the reliability of the cardiovascular evaluation, particularly in cats with an HCM phenotype. Pharmacological options, in addition to Cat-Friendly handling practices, include the pre-examination administration of gabapentin, which promotes relaxation and allows clearer separation of the E and A waves (160). A parameter strongly recommended for evaluation in feline HCM, especially due to its association with CHF, is the ratio of the peak velocity of early diastolic transmitral flow (E) to the peak velocity of late diastolic transmitral flow (A). Rohrbaugh et al. (161) demonstrated that values above 1.77 showed satisfactory sensitivity and specificity for identifying the presence of CHF (AUC = 1.0), making it a parameter of particular interest in the cardiovascular assessment of cats with an HCM phenotype.

**Figure 3:**
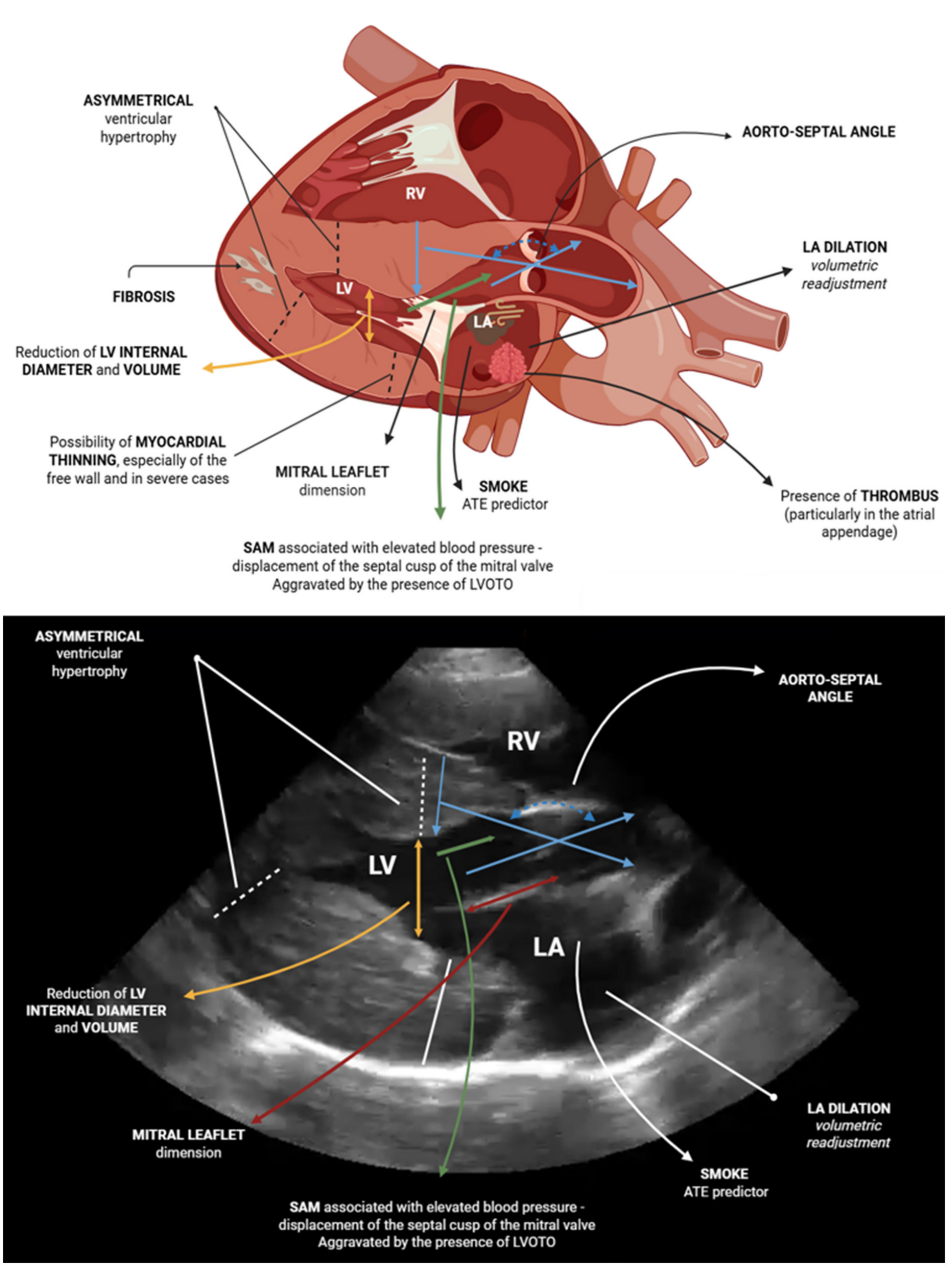
Echocardiographic findings in felines with the HCM phenotype, considering a schematic representation of the most commonly observed alterations. Created by the author with BioRender.com with Giovanni Canta figure contribution.

Given the significant atrial involvement in cats with HCM, comprehensive evaluation of the left atrium is essential. Both qualitative and quantitative approaches should be applied to assess atrial size and detect complications such as thrombus formation and spontaneous echocardiographic contrast (18). LA measurements should be obtained to calculate chamber size in long axis and the LA/Ao ratio systole and diastole (2, 148). When present, the regurgitant flow should be assessed and measured using Doppler, typically from the left atrial chamber (2). M-mode measurements acquired during systole and diastole should be recorded, focusing on LV dimensions and wall thicknesses, as these values assist in identifying structural or functional abnormalities. Additional parameters such as left atrial appendage (LAA) flow velocity provide further insight (116, 145, 150). LA/Ao ratios below 1.5 are normal (143), whereas ratios above 1.8 are risk factors for CHF and ATE (2, 95). Furthermore, LAA flow velocity >0.25 m/s (143), maximum LA diameter ≥16 mm, and SF% < 12% are echocardiographic indicators associated with increased risk of CHF and ATE (2, 95). Table 2 and Figure 3 summarizes the most frequent echocardiographic findings, and Table 3 outlines risk factors for CHF and/or ATE.

**Table 3:** High-risk echocardiographic features for the occurrence of CHF and/or ATE in cats with the HCM phenotype.

**Echocardiographic high-risk features for CHF and/or ATE**	
**Parameter**	**Description**
LA dilation	LA diameter–long axis–right parasternal four-chamber view >16 mm at end of ventricular systole and/or LA/Ao ratio >1.8
Decrease in LA fractional shortening	M-mode of the LA–short axis–percentage of systolic diameter change of the left atrium < 12%
LV systolic dysfunction	LV fractional shortening ≤ 30%
Extreme LV hypertrophy	Maximum thickness of the interventricular septum free wall or LV at end-diastole ≥9 mm
Spontaneous echocardiographic contrast	More easily visible in the LA appendage on the left parasternal view, at the plane of the base vessels—most cranial portion
Regional wall motion abnormalities	Hypokinesia of the LV free wall is generally an indicator of prior myocardial infarction in some cases
Restrictive diastolic filling pattern	Transmitral blood flow velocity: E/A >2.0
Reduced flow velocities of the LAA	Peak blood flow velocity of the LA appendage < 0.25 m/s

Information available in the studies (40, 95).

The recent study by Pons et al. (46) demonstrated that cats with thrombi and HCM associated with CHF exhibit reduced blood flow velocities in the LAA region. The presence of SEC, or “smoke,” indicates a heightened risk of thromboembolic events in these cats, requiring the administration of anti-platelet drug such as clopidogrel. Echocardiographically, spontaneous contrast appears as mobile, smoke-like echoes, particularly visible in the short-axis view at the level of the basal vessels. Felines with HCM and spontaneous contrast have been shown to exhibit reduced FS% and LA FAC, both recognized as markers of thromboembolic risk (156).

One of the largest studies in feline cardiology, the Cat Scan Study conducted by Payne et al. (3), evaluated 780 apparently healthy cats. Among the 115 cats diagnosed with HCM, fewer than 10% presented with SAM, supporting the possibility of coexistence and interdependence between HCM and SAM. Cats exhibiting SAM present with more intense murmurs, increased LV free wall thickness, and higher FS%, all associated with alterations in intracavitary pressure gradients secondary to LVOTO (29). The SAM is associated with phenotype progression and may contribute to disease worsening (31). In 2022, the Cat Scan II Study by Matos et al. (17) analyzed 107 complete datasets from apparently healthy cats with nearly 6 years of longitudinal follow-up. Findings included progressive increases in atrial dimensions and LV internal diameter during diastole, although in some cases these changes were not clinically significant. Additionally, some animals developed asymmetric hypertrophy and hypokinesia, and a proportion showed localized thinning of the LV free wall. Atrial FS% ≤ 25%, elevated ventricular FS%, and decreased LV diastolic volume were identified as predictors of HCM (17).

Systolic excursions of the mitral (MAPSE) and tricuspid annular plane (TAPSE) in cats with HCM were evaluated by Spalla et al. (147). They observed that cats with cardiac disease exhibited significantly lower values of left atrial FS%, TAPSE, tissue Doppler S' wave, and MAPSE (septal and free wall) compared to controls. Among diseased groups, cats with HCM and CHF showed more pronounced reductions in TAPSE, septal MAPSE, and free wall MAPSE compared to those with subclinical disease and healthy controls. The subclinical HCM group also exhibited significantly decreased TAPSE, septal MAPSE, and free wall MAPSE relative to controls. Moreover, survival in cats with HCM was negatively associated with increased left atrial size and LA/Ao ratio, as well as reduced left atrial FS%, TAPSE, and MAPSE (septal and free wall). Furthermore, Bach et al. (153) demonstrated the clinical relevance of MAPSE assessment by M-mode and tissue Doppler imaging-derived tissue tracking in cats with HCM, showing its association with CHF and decreased values. Another parameter recently identified by Dutton et al. (157) as valuable in echocardiographic evaluation of cats with HCM is systolic excursion of the aortic annular plane (AAPSE), with affected cats showing significantly lower values compared to healthy controls. The feasibility and practical utility of AAPSE require further evaluation and investigation regarding its diagnostic potential.

Myocardial hypokinesia and wall thinning have also been observed in cats exhibiting hypertrophic phenotype. Matos et al. (154) identified LV wall thinning in cats with HCM, findings previously reported by Payne et al. (95), who associated this phenomenon with poor clinical outcomes. Specifically, Matos et al. (154) documented myocardial segments in previously hypertrophic cats exhibiting hypokinesia and thinning [mean wall thickness before: 6.7 mm (5.8–7.7); after: 1.9 mm (1.5–2.4)]. This phenomenon predominantly affected cats in advanced stages of the disease. Felines with HCM may develop myocardial hypokinesia and wall thinning, particularly in advanced stages. These changes, documented by Payne et al. (95) and Matos et al. (154), have been associated with poor clinical outcomes.

A novel echocardiographic parameter recently investigated in cats with HCM is the aorto-septal angle and its potential association with the presence of systolic murmurs (Figure 3) (155). The authors reported that the aorto-septal angle decreases by approximately 0.55° annually, concurrent with a septal thickening of 0.11 mm, a morphological change described as a “sigmoid septum” (coefficient of determination, *R*^2^ = 0.25). An aorto-septal angle less than 120° may be linked to the presence of a murmur; notably, more than half of the felines presenting murmurs had an angle near 122° (*R*^2^ = 0.11). Furthermore, variability in the aorto-septal angle accounts for a significant proportion of isolated basal septal hypertrophy cases (*R*^2^ = 0.62). Despite these findings, the clinical utility of this measurement in cats with HCM remains to be fully validated, especially considering the modest coefficients of determination. Given that SAM may result from elevated intracavitary pressures in HCM, Seo et al. (151) evaluated the anterior mitral leaflet size as a potential marker. While human studies indicate no correlation between leaflet size and HCM development (162). Seo et al. (151) reported that cats with HCM had elongated mitral valve septal leaflets, indicating that leaflet size may serve as an independent predictor of the disease. Confirmation of this association requires further research. While these findings suggest potential diagnostic value, their clinical applicability remains uncertain and requires further investigation.

An emerging echocardiographic modality involves assessment of myocardial deformation, which allows detailed mapping of myocardial motion throughout the cardiac cycle, typically represented graphically (18). Cats with subclinical HCM show alterations in myocardial deformation along the longitudinal and axial axes, suggesting early myocardial dysfunction (149). Saito et al. (43), using strain rate imaging, identified myocardial deformation abnormalities in the longitudinal plane (epicardial and endocardial borders) and circumferential plane (primarily epicardial), accompanied by functional impairments. Alterations in myocardial deformation have been linked to the onset of congestive heart failure, with LA dilation and reduced apical LV function serving as early indicators, particularly in subclinical cats (152). More recently, Glaewketgarn and Surachetpong (19) reported significant reductions in global myocardial deformation parameters and diminished annular displacement in cats with HCM. These findings support the use of myocardial deformation metrics for diagnostic and longitudinal monitoring of myocardial function in feline HCM. Changes have been observed across longitudinal, circumferential, and axial planes, correlating with atrial dilation, reduced apical function, and early congestion. These findings suggest potential value for both diagnosis and longitudinal monitoring of myocardial function. However, methodological variability and the lack of standardized protocols limit its current applicability. Further research is required before myocardial deformation parameters can be reliably incorporated into clinical practice.

## 4 Conclusion

The findings reinforce the need for an integrated clinical, laboratory, and imaging framework to accurately identify and characterize the HCM phenotype. This phenotype, defined by concentric myocardial hypertrophy involving the LV free wall and/or interventricular septum, reduced internal diameter, atrial enlargement, and potential systemic complications, represents a progressive and deleterious cardiac condition. The broad clinical spectrum observed in affected cats leads to heterogeneous results across diagnostic modalities, emphasizing the importance of linking complementary assessments to achieve diagnostic precision. Accurate diagnosis extends beyond confirming disease presence, as it directly informs therapeutic planning and longitudinal management. A comprehensive approach should combine laboratory testing, including biomarkers of myocardial injury and function, with imaging modalities, with echocardiography remaining the cornerstone. Continued research into advanced diagnostic strategies is essential to improve understanding of the HCM phenotype and enhance diagnostic accuracy. Refinement of diagnostic criteria, phenotypic classification, staging systems, and imaging parameters will enable clinicians to make more targeted therapeutic decisions and ultimately improve outcomes in feline patients.
